# High Screen Exposure and Its Association With Physical and Mental Well-Being Among School-Going Children and Adolescents in Bangladesh: Cross-Sectional Study

**DOI:** 10.2196/73524

**Published:** 2026-05-04

**Authors:** Shahria Hafiz Kakon, Tanjir Rashid Soron, Mohammad Sharif Hossain, Biplob Hossain, Fahmida Tofail, Rashidul Haque

**Affiliations:** 1 icddr,b, 68, Shaheed Tajuddin Ahmed Sarani. Mohakhali, Dhaka, 1212, Bangladesh, 880 1726428760; 2 Tele psychiatry Research and Innovation Network Ltd, Dhaka, Bangladesh

**Keywords:** screen time, high exposure, Strengths and Difficulties Questionnaire, Pittsburgh Sleep Quality Index, Development and Well-Being Assessment scale, Bangladesh

## Abstract

**Background:**

In Bangladesh, as well as throughout the world, children’s screen time has significantly increased. Children spend a lot of time on the internet and digital screens for entertainment, education, and communication, which has increased their daily screen time. However, the potential detrimental impacts of excessive screen time on children’s mental, physical, and social health have drawn attention.

**Objective:**

This study aimed to explore the effect of high exposure to screens on the health and mental well-being of school-going children and adolescents in Dhaka, Bangladesh.

**Methods:**

This cross-sectional descriptive study was conducted from July 2022 to June 2024. A total of 420 school-going children and adolescents aged 6 to 14 years were enrolled from 3 English-language and 3 Bangla-language schools in Dhaka using a stratified random sampling technique. Anthropometric measurements, a semistructured questionnaire, and the Pittsburgh Sleep Quality Index, the Development and Well-Being Assessment scale, and the Strengths and Difficulties Questionnaire, all of which were validated in Bangla, were used to gather data. We considered students who were exposed to screens for less than 2 hours a day as the low-exposure group and those who were exposed for more than 2 hours a day as the high-exposure group.

**Results:**

A total of 83.3% (350/420) of the students were in the high-exposure group, and their average screen time per day was 4.6 (SD 2.3) hours. Eye problems were reported by 35.7% (150/420) of the students, and a significant difference was found between the low- and high-exposure groups. In total, 96% (144/150) of the students with eye problems were from the high-exposure group, whereas 4% (6/150) were from the low-exposure group. Headaches were reported by 80% (336/420) of the students, and they were common in the high-exposure group (279/336, 83%). Moreover, students from the high-exposure group had a short duration and poor quality of sleep (mean 7.3, SD 1.4 hours), which was statistically significant. Furthermore, obesity was more predominant in the high-exposure group (*P*<.001). Our study revealed that, overall, 31% (130/420) of the students had at least one mental health problem and 9.8% (41/420) had more than one mental health problem using the Development and Well-Being Assessment scale, and mental health problems were greater in the high-exposure group than the low-exposure group. Although behavioral problems such as conduct issues (119/420, 28.3%) and peer difficulties (121/420, 28.8%) were observed among the participants, no statistically significant difference was found between the 2 groups.

**Conclusions:**

A collaborative and coordinated multistage approach is essential to create effective and acceptable guidelines and policies for the optimum and positive use of digital screens for the children of Bangladesh. Further prospective studies on a larger scale can be conducted to determine the impacts of screen time on aspects of health.

## Introduction

The increasing availability and accessibility of digital devices have significantly influenced children’s lifestyles worldwide. Screen time, defined as the amount of time spent using devices such as televisions, computers, tablets, and smartphones, has become an integral part of modern childhood [1]. While moderate and purposeful screen use can offer educational benefits and entertainment, excessive and uncontrolled screen time is emerging as a major public health concern due to its potential negative impact on physical, mental, and social well-being, especially among school-going children. One of the primary concerns related to excessive screen time is its contribution to a sedentary lifestyle. Screen-based activities often replace physical activities, leading to decreased energy expenditure and increasing the risk of childhood obesity. Studies have demonstrated a strong correlation between prolonged screen time and higher BMI in children, with television viewing and digital gaming being notable contributors [2]. Furthermore, prolonged inactivity during screen use negatively affects cardiovascular and metabolic health, increasing the likelihood of developing conditions such as insulin resistance, hypertension, and dyslipidemia later in life [3]. Beyond weight-related issues, excessive screen time has been associated with musculoskeletal problems, such as poor posture and repetitive strain injuries. Extended use of handheld devices and computers can lead to neck, shoulder, and back pain, as well as long-term spinal alignment issues in growing children [4]. Sleep disturbances represent another critical concern in the context of excessive screen time. Exposure to the blue light emitted by screens, especially in the evening, can suppress melatonin production, disrupting circadian rhythms and delaying sleep onset. Insufficient and poor-quality sleep not only affects physical growth and immune function but also impairs cognitive performance and emotional regulation [5].

The implications of excessive screen time are not confined to physical health; it also significantly affects mental health and well-being. Research has indicated that more screen use is linked to higher rates of anxiety, depression, and emotional dysregulation among children and adolescents [6]. Higher durations of screen use are associated with increased risks of internalizing feelings such as sadness and withdrawal, as well as externalizing problems, including aggression and irritability [7]. The relationship between screen time and mental health is complicated, and it is frequently influenced by the context and goal of screen use. For example, passive activities such as binge-watching television are more strongly linked to adverse outcomes than active activities such as engagement with educational content or video calls that promote meaningful interactions [8]. The influence of these negative factors can cumulatively impact academic outcomes and overall health tremendously [9,10].

The social dimensions of screen time are equally important to consider. Excessive engagement with digital devices often reduces opportunities for face-to-face interactions, which are critical for developing communication and interpersonal skills during formative years. Prior studies suggest that excessive screen time often comes at the expense of time spent engaging in family activities, playing with peers, or participating in community events. Essential social skills such as empathy, communication, and conflict resolution—all of which are best acquired through face-to-face interaction—may be hampered by changes in behavior. Children who spend prolonged periods on screens may struggle with interpreting nonverbal cues and establishing meaningful connections, ultimately affecting their ability to build and sustain relationships [11]. Furthermore, exposure to inappropriate or violent content can normalize aggression and antisocial behaviors, further impairing social health outcomes [12].

The responsible use of technology plays a crucial role in children’s education, creativity, communication, and skill development [13]. Certain educational programs can positively impact children, especially those from disadvantaged backgrounds [13]. However, excessive screen time can lead to neurobiological stimulation, affecting the hypothalamic-pituitary-adrenal axis and dopaminergic pathways, which are linked to reward processing [14]. Recognizing these risks, the World Health Organization (WHO) recommends no screen time for children under 2 years, a maximum of 1 hour a day for those aged 2 to 5 years, and less than 2 hours a day for children over 5 years [15].

However, a survey in Bangladesh found that 18.9% of children use screens for more than 6 hours daily and 48% do so for approximately 4 hours. Factors such as rapid urbanization, lack of recreational spaces, and safety concerns contribute to increased indoor activities, pushing children toward excessive screen use. With digital media becoming a central part of children’s lives, it is crucial to understand its impact on their physical, mental, and social well-being. Thus, this study aims to examine the association between screen time and children’s health, focusing on patterns of use associated with psychological challenges, to inform policies and interventions that promote balanced screen use and healthier lifestyles among school-going children in Dhaka, Bangladesh.

## Methods

### Study Design and Participants

This descriptive cross-sectional study was carried out from July 2022 to June 2024. Six schools were purposively chosen from the list of schools in the Dhaka North City Corporation and Dhaka South City Corporation. Following a screening process based on the students’ exposure to digital screens and viability, 3 English-language and 3 Bangla-language schools were chosen. After obtaining permission from the school authority, a public notice was posted on the notice boards of the schools requesting students and their parents to schedule a meeting with the person conducting the research. Students who met the inclusion criteria—age between 6 and 14 years (grade 2 to grade 8) and having or not having access to gadgets such as portable gaming devices, smartphones, tablets, televisions, PCs, laptops, and gaming consoles—were chosen for the study. Following a thorough explanation of the study and provision of parents’ informed written agreement, both eligible students and their parents were invited to participate. Students who had psychological impairments, including depression, anxiety, or attention-deficit/hyperactivity disorder, that impacted their well-being or who had certain preexisting mental health disorders were excluded.

We recruited 420 students in total, 70 (16.7%) from each school and 10 (2.4%) from each grade level (2‐8), using a stratified random sampling method. The 420 participants were divided into 2 groups: one group had 70 (16.7%) students who spent less than 2 hours a day in front of devices, and the other group included 350 (83.3%) students who spent more than 2 hours a day in front of devices. The WHO and other international health regulatory agencies established the cutoff of less than 2 hours a day for children older than 5 years [15]. To collect data at the school premises, a trained team consisting of 2 field research assistants and 1 field research supervisor was involved.

### Data Collection Instruments

#### Overview

A semistructured pretested questionnaire was developed for both students and parents, and a pretest of the questionnaire was conducted on the field site on 30 nonstudy students. We conducted face-to-face interviews with parents and students at the school. It usually took 30 minutes for the students, and the interviews with the parents lasted approximately 60 minutes. We gathered information from parents about their children’s screen time, digital screen use patterns, and sociodemographic factors. Additionally, using a self-reported questionnaire, information on typical physical health issues such as headaches, back discomfort, neck pain, and eye difficulties was gathered from the students. The comments from students about their physical health were first divided into 4 categories: “No,” “Sometimes,” “Often,” and “Most of the time.” These groupings were then merged into 2 broader categories: “No” and “Yes,” where “Yes” included the responses “Sometimes,” “Often,” and “Most of the time.”

The mental health status of the students was measured using the standardized Bangla-validated Development and Well-Being Assessment (DAWBA) scale. Social behavior and emotional problems were measured using the Bangla-validated Strengths and Difficulties Questionnaire (SDQ). Finally, the quality of sleep was measured using the Bangla-validated Pittsburgh Sleep Quality Index (PSQI). The standard pre-established parameters that apply to a healthy child were compared to the health metrics obtained from each scale.

#### Anthropometric Measurements

Students’ height and weight were measured by a trained field research assistant, converted to *z*-scores, and then grouped based on the WHO BMI-for-age growth chart’s cutoff points for those aged 5 to 19 years. These measurements were standardized before and during data collection. All BMI values, which were calculated by dividing the student’s body weight by the square of their height, were categorized using the WHO cutoff criteria. According to the WHO BMI chart, the following categories apply to both boys and girls aged 6 to 14 years: underweight (<15 kg/m^2^; –1 SD), normal weight (15‐25 kg/m^2^), overweight (25‐30 kg/m^2^; +1 SD), obese (30‐40 kg/m^2^; +2 SD), and severely obese (>40 kg/m^2^; +4 SD) [16]. Three consecutive measures were taken, and the averaged value was used during data analysis.

#### SDQ Measure

The SDQ is a brief behavioral screening tool for students, where parents rate their children’s psychological traits over the previous 6 months. It includes 5 categories: emotional symptoms, conduct issues, hyperactivity and attention deficiency, peer relationship problems, and prosocial behavior, each scored on a 3-point scale (0‐2). The total score (maximum of 40) reflects overall behavioral challenges, whereas prosocial behavior is scored separately (maximum score of 10). The SDQ was translated into Bangla and validated in Bangladesh by Mullick and Goodman [17] in 2001.

#### PSQI Measure

The Bangla PSQI was used to evaluate overall sleep quality. Students completed this self-reported questionnaire, which assigns scores from 0 to 3 across various subcategories, including subjective sleep quality, sleep duration, sleep latency, habitual sleep efficiency, disruptions, sleep medicine use, and dysfunction during the day. A score of 5 or more indicates poor sleep quality, and the maximum score is 21 [18].

#### DAWBA Measure

To evaluate mental health problems, we used the DAWBA, a widely used questionnaire among children and adolescents aged 5 to 17 years. The structured questions and open-ended verbatim accounts were assessed by DAWBA raters to generate *International Classification of Diseases, 10th Revision*, or *Diagnostic and Statistical Manual of Mental Disorders, Fourth Edition*, psychiatric diagnoses. In this analysis, we used the Bangla version of the DAWBA that was validated by Mullick and Goodman [19] in 2005.

### Statistical Analysis

Data were imported into Microsoft Excel for statistical analysis, cleaned, and then exported into SPSS (version 20; IBM Corp) and Stata (version 15.1; StataCorp). Two main groups were formed among the participants: those with high exposure (>2 hours a day) and those with low exposure (<2 hours a day). A comparative analysis was then carried out across several variables, such as demographic characteristics, screen time, and health metrics. For categorical data, chi-square tests for independence were used to evaluate differences between groups, and for continuous data with nearly normal distributions, an unpaired 2-tailed *t* test for differences between proportions was used. A significance threshold of a *P* value of less than .05 was used to assess statistical significance.

### Ethical Considerations

This study was approved by the institutional review board of icddr,b (protocol PR-22002). Confidentiality and anonymity were maintained throughout the study. Parental permission was obtained through informed written consent for all participants aged 6 to 14 years. Additionally, assent was obtained from students aged between 11 and 14 years. Every respondent received information in Bangla on their rights regarding their voluntary involvement in the study and their ability to leave the interview at any point while it was being conducted.

## Results

### Sociodemographic Characteristics of the Study Participants

The study included 420 students based on screening criteria, of whom 350 (83.3%) were categorized in the high-exposure group (using digital devices for more than 2 hours daily) and 70 (16.7%) were categorized in the low-exposure group. The students were aged between 6 and 14 years, with a mean age of 10.9 (SD 1.9) years, with just over half of them (n=213, 50.7%) being male. The average daily screen time of the parents was 3.9 (SD 2.7) hours, and the average daily screen time of the students was 4.6 (SD 2.3) hours according to their own statement. Table 1 presents detailed information on the sociodemographic characteristics of the study population.

**Table 1.** Sociodemographic characteristics of the study participants (N=420).

Characteristic	Overall	Low exposure (<2 h/d; n=70)	High exposure (>2 h/d; n=350)
Students’ age (y), mean (SD)	10.9 (1.9)	11.2 (1.8)	10.9 (1.9)
Students’ sex, n (%)			
Male	213 (50.7)	35 (50)	178 (58.9)
Female	207 (49.3)	35 (50)	172 (49.1)
Screen time–based information, mean (SD)			
Time spent by parents (h/d)	3.9 (2.7)	1.7 (1.5)	4.3 (2.7)
Time spent by the students (parents’ statement; h/d)	4.5 (2.4)	1.1 (0.5)	5.1 (2.1)
Time spent by the students (students’ statement; h/d)	4.6 (2.3)	1.2 (0.6)	5.2 (1.9)

### Prevalence of Physical Symptoms by Screen Exposure Level

The prevalence of physical symptoms among study participants with high and low exposure is shown in Table 2. Of the 420 students included in the study, 150 (35.7%) had eye problems. Of these 150 affected students, 144 (96%) belonged to the high-exposure group, whereas only 6 (4%) were from the low-exposure group, showing a statistically significant association (*P*<.001). Among all participants, 80% (336/420) reported headaches, 65.7% (276/420) experienced indigestion, and 81.2% (341/420) reported abdominal pain. Additionally, 45.7% (192/420) reported neck pain, 31.9% (134/420) reported back pain, and 48.6% (204/420) reported changes in appetite. These symptoms were more prevalent in the high-exposure group, although the differences were not statistically significant. Hearing difficulties (14/420, 3.3%) and diabetes (4/420, 1%) were the least frequently reported symptoms among the high-exposure group.

**Table 2.** Prevalence of physical symptoms among study participants in the high- and low-exposure group (N=420).

Characteristic	Overall, n (%)	Low exposure (<2 h/d; n=70), n (%)	High exposure (>2 h/d; n=350), n (%)	*P* value
Eye problems				.001
No	270 (64.3)	64 (91.4)	206 (58.9)	
Yes	150 (35.7)	6 (8.6)	144 (41.1)	
Hearing difficulties				.33
No	406 (96.7)	69 (98.6)	337 (96.3)	
Yes	14 (3.3)	1 (1.4)	13 (3.7)	
Indigestion				.78
No	144 (34.3)	25 (35.7)	119 (34)	
Yes	276 (65.7)	45 (64.3)	231 (66)	
Headache				.74
No	84 (20)	13 (18.6)	71 (20.3)	
Yes	336 (80)	57 (81.4)	279 (79.7)	
Neck pain				.43
No	228 (54.3)	41 (58.6)	187 (53.4)	
Yes	192 (45.7)	29 (41.4)	163 (46.6)	
Abdominal pain				.29
No	79 (18.8)	10 (14.3)	69 (19.7)	
Yes	341 (81.2)	60 (85.7)	281 (80.3)	
Back pain or any other musculoskeletal problem				.08
No	286 (68.1)	54 (77.1)	232 (66.3)	
Yes	134 (31.9)	16 (22.9)	118 (33.7)	
Diabetes				.37
No	416 (99)	70 (100)	346 (98.9)	
Yes	4 (1.0)	0 (0)	4 (1.1)	
Any changes in appetite				.60
No	216 (51.4)	38 (54.3)	178 (58.9)	
Yes	204 (48.6)	32 (45.7)	172 (49.1)	

### Screen Time and Obesity by Level of Exposure

**Figure 1.**
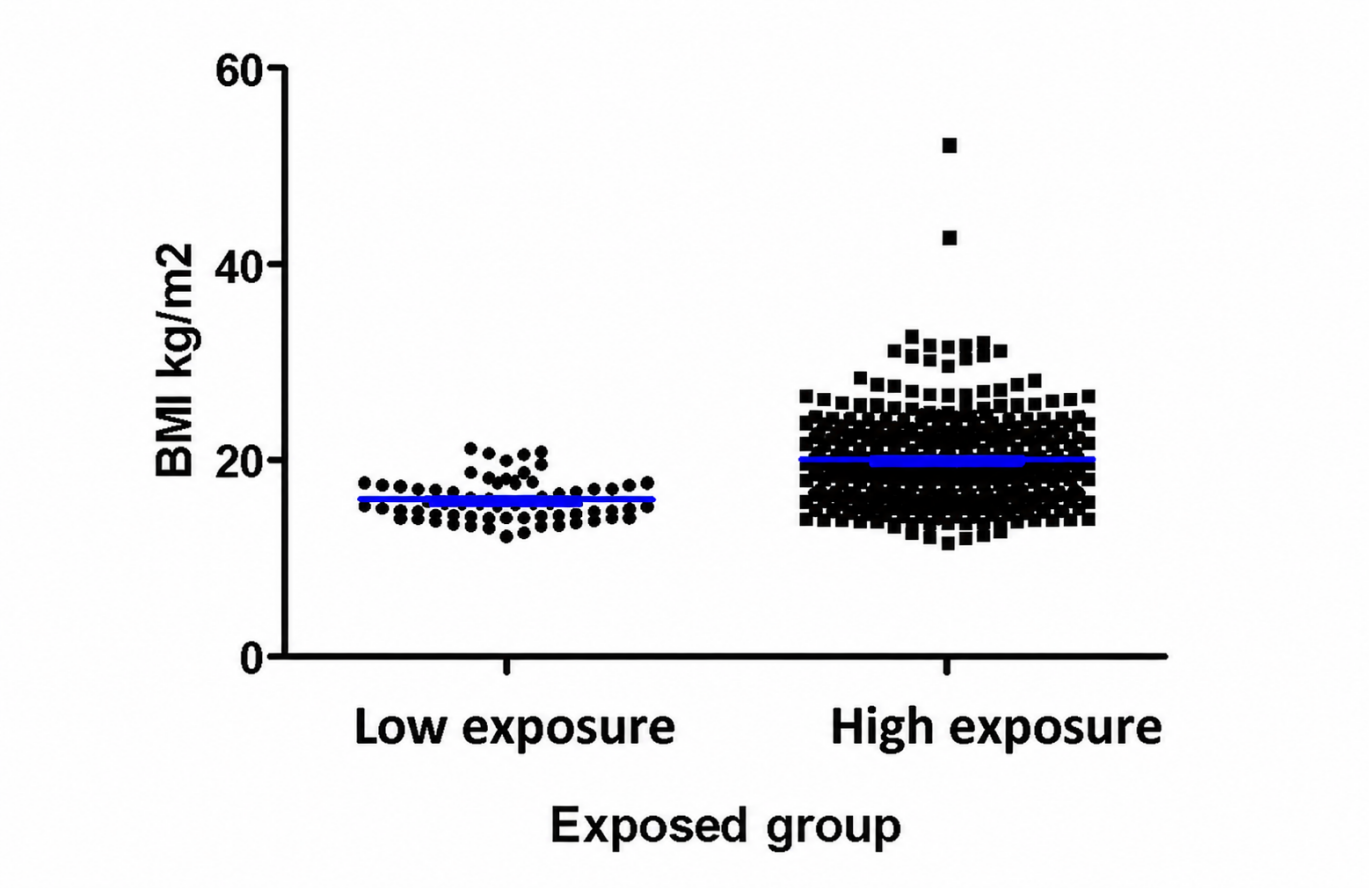
Screen time and obesity in the low- and high-exposure groups.

The mean BMI for the study population was 19.3 (SD 4.7) kg/m^2^ (Figure 1). Students with high exposure to digital screens had an average BMI of 20.0 (SD 4.7) kg/m^2^ compared to 15.96 (SD 2.08) kg/m^2^ in the low-exposure group, which was statistically significant (*P*<.001). Additionally, students categorized as being overweight (42/420, 10.1%), obese (16/420, 3.8%), or severely obese (4/420 (0.9%) were predominant in the high-exposure group (*P*<.001) compared to the low-exposure group.

### Association Between Screen Time and Behavior by Exposure Level (SDQ)

The SDQ analysis revealed that, of the 420 students, 119 (28.3%) exhibited conduct problems, 121 (28.8%) had peer relationship problems, 66 (15.7%) reported emotional issues, 73 (17.4%) experienced hyperactivity, and 28 (6.7%) exhibited prosocial behaviors. Overall, most students fell within the normal range for these domains. However, among students classified as borderline or abnormal, most were in the high-exposure group. For example, in the peer relations domain, 28.8% (121/420) of the students were classified as abnormal, of whom 87.6% (106/121) were in the high-exposure group, whereas only 12.4% (15/121) were in the low-exposure group. Despite these trends, none of the SDQ domains showed statistically significant associations with screen time. Table 3 illustrates the relationship between screen time and the SDQ among the low- and high-exposure groups.

**Table 3.** Correlation between child behavior and screen time in the low-exposure and high-exposure groups based on the Strengths and Difficulties Questionnaire (SDQ).

SDQ domains		Overall (n=420), n (%)	Low exposure (<2 h/d) (n=70), n (%)^a^	High exposure (>2 h/d) (n=350), n (%)^a^	*P* value
Emotional problems					>.99
	Normal	354 (84.3)	59 (16.7)	295 (83.3)	
	Borderline/abnormal	66 (15.7)	11 (16.7)	55 (83.3)	
Conduct problems					.96
	Normal	310 (71.7)	50 (16.6)	251 (83.4)	
	Borderline/abnormal	119 (28.3)	20 (16.8)	99 (83.2)	
Hyperactivity/inattention					.27
	Normal	347 (82.6)	61 (17.6)	286 (82.4)	
	Borderline/abnormal	73 (17.4)	9 (12.3)	64 (87.7)	
Peer relation problems					.13
	Normal	299 (71.2)	55 (18.4)	244 (81.6)	
	Borderline/abnormal	121 (28.8)	15 (12.4)	106 (87.6)	
Prosocial behavior					.86
	Normal	392 (93.3)	65 (16.6)	327 (83.4)	
	Borderline/abnormal	28 (6.7)	5 (17.9)	23 (82.1)	

a Percentages in these columns are calculated with the value in the “Overall” column as the denominator.

### Association Between Screen Time and Sleep Quality by Exposure Level (PSQI)

The correlation between sleep and screen time in the low- and high-exposure groups according to the PSQI is shown in Table 4. The overall average sleep latency for all participants was 20.3 (SD 14.7) minutes, with the high-exposure group showing significantly longer latency (mean 21.1, SD 15.4 minutes) than the low-exposure group (mean 16.3, SD 10.1 minutes; *P*=.01). Additionally, the average sleep duration was significantly shorter in the high-exposure group (mean 7.3, SD 1.4 hours) than in the low-exposure group (mean 9.0, SD 1.4 hours; *P*<.001), indicating poorer sleep quality among students with higher screen exposure. An evaluation of the PSQI components revealed significant differences in sleep duration between the 2 groups (*P*<.001). Other components, such as breathing difficulties, bad dreams, sleep disturbances, and daytime dysfunction, were also more prevalent in the high-exposure group than in the low-exposure group.

**Table 4.** Correlation between sleep and screen time in the low- and high-exposure groups according to the Pittsburgh Sleep Quality Index (PSQI).

PSQI variable	Low exposure (<2 h/d; n=70)	High exposure (>2 h/d; n=350)	*P* value
Sleep latency (min), mean (SD)	16.3 (10.1)	21.1 (15.4)	.01
Sleep duration (h), mean (SD)	9.0 (1.4)	7.3 (1.4)	<.001
Waking up in the middle of the night, n (%)			.01
Not during the previous month	32 (45.7)	182 (52.0)	
Less than once a week	16 (22.9)	103 (29.4)	
Once or twice a week	18 (25.7)	36 (10.3)	
3 or more times a week	4 (5.7)	29 (8.3)	
Sleep duration (h), n (%)			<.001
>7	65 (92.9)	172 (49.1)	
6-7	5 (7.1)	118 (33.7)	
5-6	0 (0)	44 (12.6)	
<5	0 (0)	16 (4.6)	

### Association Between Screen Time and Mental Health by Exposure Level

The correlation between student mental health and screen time in the low- and high-exposure groups according to the DAWBA scale is shown in Table 5. The DAWBA scale revealed that 31% (130/420) of the students reported at least one mental health issue, including specific phobia, generalized anxiety, depression, deliberate self-harm, hyperactivity, awkward behavior, and troublesome behavior. Additionally, 9.8% (41/420) of the students were identified as having more than one mental health problem. Notably, of the students with at least one mental health issue, 86.9% (113/130) were from the high-exposure group (screen use exceeding 2 hours per day). Similarly, among those with more than one mental health problem, 92.7% (38/41) belonged to the high-exposure group. The association between screen time and mental health problems showed borderline statistical significance, with a *P* value of .05.

**Table 5.** Correlation between student mental health and screen time in the low- and high-exposure groups according to the Development and Well-Being Assessment scale (N =420)^a^.

	Overall, n (%)	Low exposure (<2 h/d; n=70), n (%)	High exposure (>2 h/d; n=350), n (%)	*P* value
No mental health problems	249 (59.3)	50 (71.4)	199 (56.9)	.05
At least one mental health problem	130 (31.0)	17 (24.3)	113 (32.3)	.05
More than one mental health problem	41 (9.8)	3 (4.3)	38 (10.9)	.05

a Mental health problems assessed: specific phobia, generalized anxiety, depression, deliberate self-harm, hyperactivity, awkward behavior, and troublesome behavior.

## Discussion

### Principal Results and Comparison With Prior Work

We found that a large majority of students (350/420, 83.3%) were exposed to digital screens for more than 2 hours per day and that higher screen exposure was strongly associated with poorer health outcomes. Nearly all students with eye problems (144/150, 96%) belonged to the high-exposure group (*P*<.001). Students with high screen time also had significantly higher BMI, with overweight (42/420, 10.1%) and obese (16/420, 3.8%) status concentrated almost entirely in this group (*P*<.001). In addition, high screen exposure was linked to poorer sleep, characterized by longer sleep latency and significantly shorter sleep duration (mean 7.3, SD 1.4 hours vs mean 9.0, SD 1.4 hours; *P*<.001). Finally, almost one-third of the students (130/420, 31%) had at least one mental health problem, with the vast majority (113/130, 86.9%) occurring among students with high screen exposure (*P*=.05).

In this study, the mean age of the participants was 10.9 (SD 1.9) years, with 49.3% (207/420) of the students being female. In a similar study, participants had a mean age of 10.9 (SD 1.16) years, and 47.7% were female [20]. Our study also found that the mean screen time per day was 4.6 (SD 2.3) hours from the students’ perspective, which was similar to the parents’ perspective (mean 4.5, SD 2.4 hours). Seguin et al [21] reported similar findings, which were a mean screen time of 5.9 (SD 3.4) hours per day. In addition, a systematic review showed that, overall, screen time among children aged 6 to 10 years and adolescents increased by 1.4 hours per day and 0.91 hours per day (in the year 2020-2021), respectively, compared to the pre–COVID-19 era [22]. The lockdown regimen during the pandemic brought a huge change to screen time duration and patterns in the daily routines of children. With regard to physical symptoms, 80% (336/420) of students in our study were found to have headaches. A recent study reported that there was a statistically significant association between increased screen time duration and headache, as well as dry eyes (*P=*.04 and *P*=.02, respectively) [23].

We found statistically significant associations between screen time duration and BMI. Similar findings were reported in Austria, where screen time was significantly associated with a higher BMI (*P*=.002) [24]. In contrast, a study in Pakistan demonstrated that there was no association between mean screen time and BMI (*P*=.64) [25]. The disparities may be attributable to various confounding factors such as food traditions and lifestyle.

In this study, as per the SDQ, in terms of prosocial behaviors, it was observed that only 6.7% (28/420) of the students were in the category of abnormal scores overall, with 7.1% (5/70) and 6.6% (23/350) in the lower- and higher-exposure groups, respectively. However, a study conducted on rural Chinese school-going children revealed that 10.5% of the participants belonged to the abnormal prosocial behavior category [26]. These differences may be due to other mental health problems as the community where the aforementioned study was conducted may have had various familial conflicts with lower levels of social support. In addition, previous research does show that screen time above the threshold is linked with increased abnormalities in behavior.

Regarding sleep habits and patterns, we found that duration of sleep was associated with screen time duration (*P*<.001). In a previous study in India, it was observed that both sleep efficiency and sleep duration were associated with screen time duration (*P*=.003 and *P*=.001, respectively) [27]. Another study reported that, among children who were exposed to screens for more than 2 hours a day, 61.8% had a PSQI score lower than 5 [28]. Multiple mechanisms suggest that screen time and media use are linked to sleep disturbances. There are several stimulating programs and contents that increase the activity level of the nervous system, inhibiting relaxation and leading to anxiety and/or resulting in raised alertness and trouble falling asleep [29].

In the last decade, changes in screen use patterns and duration, type of content, type of device used, and parental monitoring have occurred. As a result, various types of studies have assessed the relationship between multiple aspects of screen time and/or device use and different facets of physical, mental, and social health in children, adolescents, and young adults, as well as academic performance outcomes and overall well-being. A study published in 2024 among children aged 6 to 10 years on screen addiction revealed that the group with high screen exposure showed statistically significant differences in screen addiction, distraction, and sedentary factors (*P*<.05) [30]. Another study reported that children who had greater exposure to screens, along with shorter sleep duration and lower physical activity levels, had poorer health-related quality of life [31]. In addition, a relevant study illustrated that children with exposure to screens for more than 2 hours per day had an increased likelihood of reported behavioral problems and poorer vocabulary [32].

It is worth mentioning that screen use widely varies across contexts. For instance, for academic purposes, screen time can exceed the recommended duration on certain days. This can be observed in the case of meeting deadlines or acquiring knowledge, where the screen is the only medium available. Another point to be kept in mind is the type of media being “overused” [33]. All in all, the context, platform, regimen, and secular trends should play a role in determining screen time duration.

### Limitations

First, the 6 schools included in this study (3 in Bangla and 3 in English) may not adequately reflect the wider differences in resources between urban and rural environments. Second, there may have been recall bias because data on technological interactions were gathered through in-person interviews. Future studies on the effects of screen time ought to consider a wider range of settings and demographics. Study designs other than cross-sectional should be used, and extensive analyses involving other covariates should be conducted.

### Strengths

Despite the aforementioned limitations, the study’s strength lies in its innovative examination of the association between varying levels of screen exposure (low vs high) and the physical, mental, and social well-being of Bangladeshi students, particularly given that children with low exposure to digital screens are increasingly difficult to find. Furthermore, this study offers an insightful comparative analysis because it included students from Bangla- and English-language schools. The findings can inspire and inform parents, legislators, and educational authorities, emphasizing supporting students’ healthier use of digital devices and more balanced lifestyles. More investigation is needed, possibly using longitudinal or experimental designs to examine causal relationships.

### Conclusions

The findings of this study improve our knowledge of strategies to lessen the detrimental effects that excessive digital screen time has on children’s mental health and well-being. Educators, parents, legislators, and children themselves must all be involved to create effective screen use guidelines. By holding workshops and seminars, schools can effectively teach children about responsible screen use and enforce a balanced screen time policy. To improve their children’s well-being, parents must be aware of the dangers of excessive screen time as well as the advantages of active supervision. Accessible mental health resources, such as support groups and counseling, can also assist students in reducing the stress and anxiety associated with screen time. Additional research must be conducted to promote the development of comprehensive guidelines for screen time.

## Abbreviations

DAWBA: Development and Well-Being Assessment
PSQI: Pittsburgh Sleep Quality Index
SDQ: Strengths and Difficulties Questionnaire
WHO: World Health Organization
